# Frequent genomic imbalances suggest commonly altered tumour genes in human hepatocarcinogenesis

**DOI:** 10.1054/bjoc.2001.1963

**Published:** 2001-09

**Authors:** F Niketeghad, H J Decker, W H Caselmann, P Lund, F Geissler, H P Dienes, P Schirmacher

**Affiliations:** Institute of Pathology, University of Cologne, Joseph Stelzmann Str. 9, Cologne, D-50931, Germany; III. Medical Department, University of Mainz, Langenbeckstr. 1, Mainz, D-55101, Germany; Department of General Internal Medicine, University of Bonn, Sigmund Freud Str. 25, Bonn, D-53105, Germany; Department of Surgery, University of Leipzig, Germany

**Keywords:** hepatocellular carcinoma, oncogenes, tumour suppressor genes, hepatocarcinogenesis, comparative genomic hybridization, FISH

## Abstract

Hepatocellular carcinoma (HCC) is one of the most frequent-occurring malignant tumours worldwide, but molecular changes of tumour DNA, with the exception of viral integrations and p53 mutations, are poorly understood. In order to search for common macro-imbalances of genomic tumour DNA, 21 HCCs and 3 HCC-cell lines were characterized by comparative genomic hybridization (CGH), subsequent database analyses and in selected cases by fluorescence in situ hybridization (FISH). Chromosomal subregions of 1q, 8q, 17q and 20q showed frequent gains of genomic material, while losses were most prevalent in subregions of 4q, 6q, 13q and 16q. Deleted regions encompass tumour suppressor genes, like RB-1 and the cadherin gene cluster, some of them previously identified as potential target genes in HCC development. Several potential growth- or transformation-promoting genes located in chromosomal subregions showed frequent gains of genomic material. The present study provides a basis for further genomic and expression analyses in HCCs and in addition suggests chromosome 4q to carry a so far unidentified tumour suppressor gene relevant for HCC development. © 2001 Cancer Research Campaign http://www.bjcancer.com


*British Journal of Cancer* (2001) **85**(5), 697–704
© 2001 Cancer Research Campaign
doi: 10.1054/ bjoc.2001.1963, available online at http://www.idealibrary.com on IDEAL
http://www.bjcancer.com

### Frequent genomic imbalances suggest commonly altered tumour genes in human hepatocarcinogenesis

**F Niketeghad[1], HJ Decker[2], WH Caselmann[3], P Lund[1], F Geissler[4], HP Dienes[1] and P Schirmacher[1]**

[1]Institute of Pathology, University of Cologne, Joseph Stelzmann Str. 9, D-50931 Cologne, Germany; [2] III. Medical Department, University of Mainz, Langenbeckstr. 1, D-55101 Mainz, Germany; [3]Department of General Internal Medicine, University of Bonn, Sigmund Freud Str. 25, D-53105 Bonn, Germany; and [4]Department of Surgery, University of Leipzig, Germany

**Summary** Hepatocellular carcinoma (HCC) is one of the most frequent-occurring malignant tumours worldwide, but molecular changes of tumour DNA, with the exception of viral integrations and p53 mutations, are poorly understood. In order to search for common macro-imbalances of genomic tumour DNA, 21 HCCs and 3 HCC-cell lines were characterized by comparative genomic hybridization (CGH), subsequent database analyses and in selected cases by fluorescence in situ hybridization (FISH). Chromosomal subregions of 1q, 8q, 17q and 20q showed frequent gains of genomic material, while losses were most prevalent in subregions of 4q, 6q, 13q and 16q. Deleted regions encompass tumour suppressor genes, like RB-1 and the cadherin gene cluster, some of them previously identified as potential target genes in HCC development. Several potential growth- or transformation-promoting genes located in chromosomal subregions showed frequent gains of genomic material. The present study provides a basis for further genomic and expression analyses in HCCs and in addition suggests chromosome 4q to carry a so far unidentified tumour suppressor gene relevant for HCC development. © 2001 Cancer Research Campaign http://www.bjcancer.com

**Keywords**: hepatocellular carcinoma, oncogenes, tumour suppressor genes, hepatocarcinogenesis, comparative genomic hybridization, FISH

Hepatocellular carcinoma (HCC) is one of the most common malignant tumours worldwide (Bosch, 1997). It has a well-defined etiology and develops almost exclusively in patients with chronic liver damage due to chronic viral infections (hepatitis B and C), metabolic liver diseases (e.g. haemochromatosis), and toxins (alcohol, aflatoxin B1) (Schirmacher and Dienes, 1999). Typically its development encompasses several decades and requires several genetic and/or epigenetic alterations to occur in affected hepatocytes in order to complete malignant transformation, but the responsible molecular changes are only rudimentarily known.

Present data suggest a frequent involvement of p53 mutations (Bressac et al, 1991; Hsu et al, 1991) as well as functional RB-1 deletions in human HCCs (Nishida et al, 1992; Murakami et al, 1991; Walker et al, 1991). Furthermore, evidence for a tumour suppressor function of the insulin-like growth factor (IGF)-II receptor in liver tumourigenesis has accumulated (Yamada et al, 1997). Growth factors, like IGF-II and transforming growth factor (TGF)-α, are known to be frequently overexpressed and support growth in an autocrine manner in HCCs (Derynck et al, 1987; Schirmacher et al, 1992; Nong et al, 1997), while the tumorigenic role of proto-oncogenes is poorly characterized. So far MYC overexpression and *ras*-gene mutations appear to be rather infrequent in human HCCs (Geissler and Gesien, 1997), in contrast to some well-characterized animal models (Sinha et al, 1988; Fourel et al, 1990; McMahon et al, 1990). Recently oncogenic mutations of the β-catenin gene have been found in 26% of human HCCs (de la Coste et al, 1998). Altogether, these data have established the relevance of several tumour genes and suggest that a number of alterations in different classes of genes are required to complete the process of hepatocarcinogenesis. The data are nevertheless biased, since they are derived from selected expression analyses. Thus there is a need for a largely unbiased screening to detect genetic events so far not connected with HCC development. Previous studies using loss of heterozygosity (LOH) analyses (Tsuda et al, 1990; Nishida et al, 1992; Tsuda et al, 1992; Nagai et al, 1997) were restricted by the selection of LOH markers and the fact that by this technique only the detection of potential tumour suppressor gene loci is feasible. Furthermore data from LOH analyses in HCCs vary significantly between the different studies; the resulting LOHs are widely distributed throughout the genome and have therefore not contributed significantly to the identification of tumour-relevant genes.

We have analysed well-characterized HCCs by comparative genomic hybridization (CGH) and have performed database analyses to search for potential tumour-relevant genes so far not linked to hepatocarcinogenesis encompassing the identified altered chromosomal regions. Our data show frequent gains of genomic material in chromosomes 1q, 8q, 17q and 20q as well as losses in 4q, 6q, 13q and 16q, and suggest that genes located in these chromosomal regions contribute to hepatocarcinogenesis.

*Received 2 June 2000*
*Revised 22 February 2001*
*Accepted 28 February 2001*

*Correspondence to:* P Schirmacher

### MATERIAL AND METHODS

### Tissues and cell lines

Tissues were removed by surgery, partly fixed in formalin and partly frozen immediately, and stored in pieces of less than 1 $cm^3$ at –70˚C. Tumours were histologically characterized using representative 2 µm sections obtained from formalin-fixed, paraffin-embedded tissues. Tissues used for DNA extraction and subsequent CGH analyses were especially controlled for containing more than 90% tumour tissue and the lack of significant necroses or preservation artifacts by histological analysis of corresponding frozen sections. Grading of HCCs was performed as recommended by the World Health Organization (WHO) UICC (Ishak et al, 1994).

Cell lines HEP-3B, HEP-G2, and SK-HEP1 were purchased directly from the Deutsche Sammlung für Mikroorganismen und Zellkulturen (Braunschweig, Germany) and were stored and cultured as suggested by the supplier.

### DNA-extraction and CGH

Genomic DNA was obtained from frozen tissues (tumours), cell lines or peripheral blood cells of normal persons (reference DNA) by phenol/chloroform extraction according to a standard protocol (Sambrook et al, 1989). The quality of genomic DNA was controlled by submitting unrestricted aliquots to 1% agarose gel electrophoresis prior to the labelling reaction.

For chromosomal metaphase preparations, peripheral blood lymphocytes were obtained from normal persons, cultured and stimulated with phytohaemagglutinin (1.5%; Gibco BRL, Eggenstein, Germany) in McCoy's 5A medium (Gibco BRL). After culturing for 72 h, metaphase arrest was performed with colcemid (0.3 µg/ml; Gibco BRL). All further preparations were performed according to a standard protocol (Vogel and Speit, 1985).

CGH was performed according to Lichter et al (1994) with slight modifications. Slides containing the prepared metaphases were rehydrated in graded ethanol, 0.1 × standard saline citrate (SSC), and 2 × SSC, subsequently heat stabilized in 2 × SSC at 75˚C for 30 min, denatured in 0.07 N NaOH and dehydrated in graded ethanol. Test and reference DNAs were labelled by nick translation (CGH-nick translation kit; Vysis, Downers Grove, IL, USA). Hybridization on chromosomal metaphase preparations was carried out at 37˚C (low stringency) or 55˚C (high stringency) for 3 d. After the hybridization reaction the slides were washed in 2 × SSC and 2 × SSC Tween 20 at room temperature and 1 × SSC at 75˚C.

The ratio profiles of each individual chromosome were determined using a cytovision 3.1 software (Applied Imaging, Sunderland, UK). A FITC/TRITC ratio ranging from 0.75–1.25 indicated balanced genomic material (Du Manoir et al, 1995). In most cases the complete hybridization reaction and analyses were performed twice, under low (37˚C), and high stringency (55˚C) hybridization conditions, and only those imbalances of genomic material reproducible under both conditions were accepted. Chromosomal regions showing significant frequencies of genomic alterations were searched for potential tumour-relevant genes using the OMIM-NCBI database (www.ncbi.nlm.nih.gov/omim/searchmap.html), which is currently estimated to contain more than half of the expected human genes annotated by chromosomal localization (Deloukas et al, 1998). The current analysis focussed on chromosomal regions that were frequently altered in the CGH analysis. These chromosomal regions that were screened for those genes that either had an already established or suspected function in human hepatocarcinogenesis or a known oncogenic or tumour-suppressive function, matched to the respective type of genomic imbalance and were likely to act in carcinoma cells (Figure 1).

### Interphase fluorescence in situ hybridization (FISH)

Interphase FISH was performed in selected representative cases (3) with a limited number of frequently occurring genomic imbalances in order to confirm the results of the CGH analysis. Isolated interphase nuclei were obtained according to Hopman et al (1992) except that fixation was performed in methanol/acetic acid (3:1). Hybridization reactions were performed with CEP- and LSI-DNA FISH probes (Vysis). Fluorescence microscopy and photomicroscopy were performed with a Leica DMRBE microscope (Leica, Bensheim, Germany). Hybridization was carried out simultaneously with a test probe directed against a chromosomal region showing genomic imbalance (1q: 1q12 (sat. II and III); 8: 8p11.1-q11.1 (α-sat. DNA); 13q: 13q14 (RB-1 locus)) and a reference probe derived from a region without significant alteration according to the respective CGH ratio profile (10p11.1-q11.1 (α-sat. DNA); 17p11.1-q11.1 (α-sat. DNA)). In each analysis more than 100 interphase nuclei were evaluated by fluorescence microscopy. The number of signals per nucleus were counted for each probe. Then the ratio of test probe signal/reference probe signal was determined for each signal interphase nucleus. The nuclei were then grouped according to this signal ratio.

### cDNA-arrays cDNA expression analysis was performed in tumour No. 6 containing the frequently occurring gains of genomic material at 1q and 8q (as well as X) in order to test in an unbiased manner whether the genomic imbalances were associated with alterations in expression of genes located in these chromosomal regions. Atlas Human cDNA Expression Arrays (Clontech, Palo Alto, CA, USA) were performed as recommended by the supplier.

Filter analysis and semiquantitative evaluation was performed with a Storm 840 phosphofluoroimager (Molecular Dynamics, Sunnyvale, CA, USA) using the Image QuaNT software (version 5.0; Molecular Dynamics).

### RESULTS

All HCCs that were analysed met the histological pre-evaluation criteria and resulted in well-interpretable metaphases and CGH ratio profiles. In all HCCs and cell lines with the exception of 3 HCCs, significant genomic imbalances were detected (Table 1). All cell lines showed a rather high number of genomic imbalances.

In tumour No. 6, the CGH ratio profiles showed a gain of 1q and an amplification of 8q. In FISH analyses of isolated interphase HCC nuclei with centromeric probes from both chromosomes, significant numerical over-representations of the hybridization signals for each of the test probes were detectable compared to the reference probes (Figure 1A and B, Table 2).

In tumour No. 8, among other alterations, a significant loss of 13q encompassing 13q14.1-14.2, the location of the RB-1 gene was found. Here interphase FISH also detected under-representation of 13q14.1-14.2 using a RB-1 locus-specific probe compared to a control probe from the centromeric region of chromosome 10 that appeared unaltered in the CGH ratio profile (Figure 2c, Table 2)

Overall gains of genomic material outnumbered losses in frequency (Table 1). When the genomic imbalances were sorted by chromosomal location (Figure 1, Table 1B), gains were most frequently detected in: 1q (57%, core region: 1q21-24), 8q (52%, core region: 8q24.1-24.3), 17q (29%) and 20q (29%). Losses were

*© 2001 Cancer Research Campaign*

BJOC 01-1963 697-704 20/8/01 3:25 pm Page 699

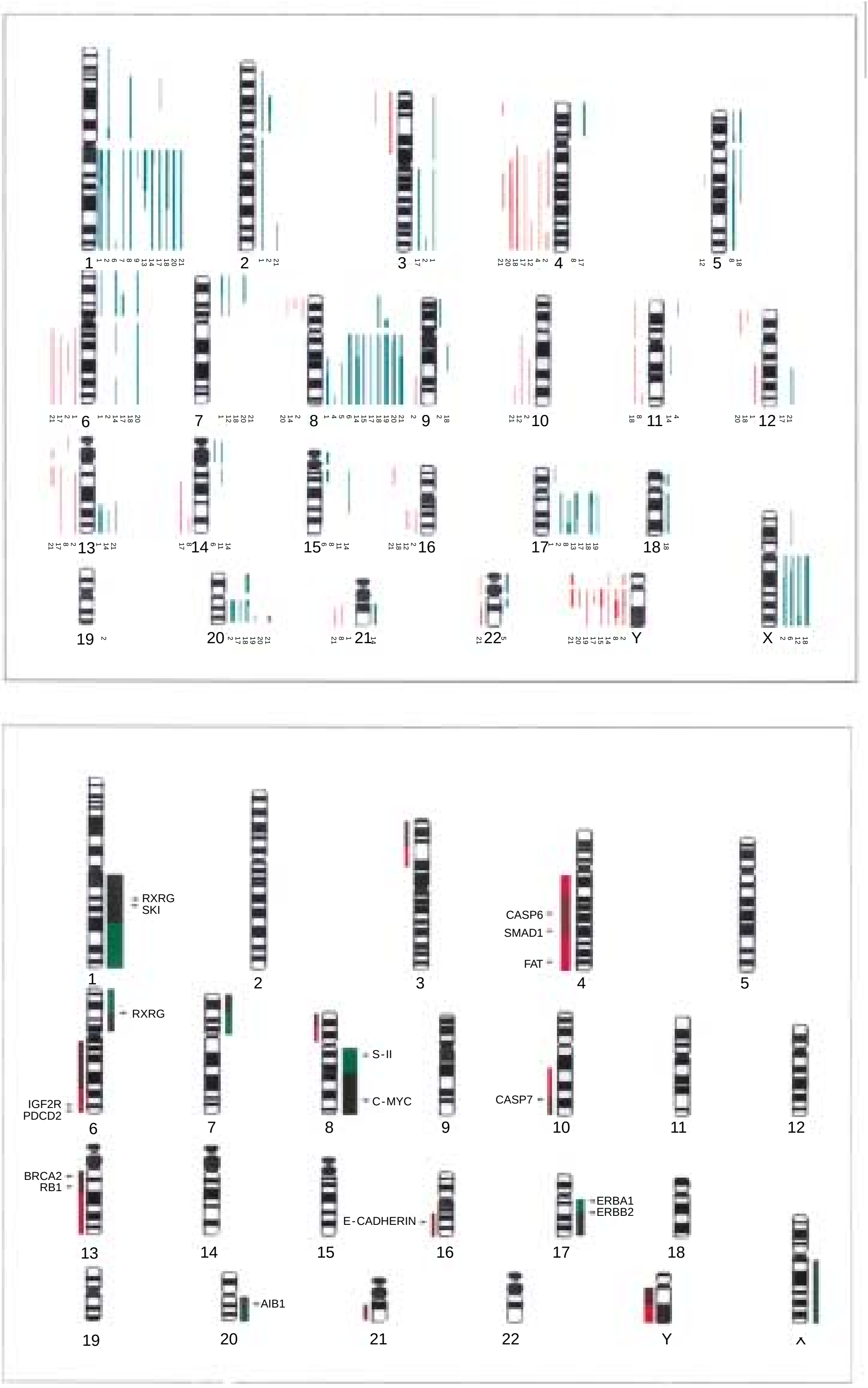

**Figure 1** Summarized display of genomic imbalances detected in 21 HCCs. (above) Gains of genomic material are shown on the right side; losses are shown on the left side of each chromosome, corresponding to the respective chromosomal regions. Intensity of the lines corresponds to the extent of gains (thin line: ratio of 1.25–1.5, thick line > 1.5). Data correspond to Table 1. (below) Localization of significant gains and losses of genomic material. Chromosomal areas with significant genomic imbalances are shown as boxes with gains located to the right and losses located to the left of the respective chromosomal region. Consensus core regions are shown as black boxes. The location of some potential tumour-relevant genes in these areas is shown (see Discussion)

less frequent but significantly altered regions were present in 4q (33%, core region: 4q22-28), 6q (19%), 13q (19%), and 16q (14%). The same alterations were also in part represented in the 3 analysed cell lines. There was no indication for certain genomic

BJOC 01-1963 697-704 20/8/01 3:25 pm Page 700

**Table 1** Genomic imbalances in HCCs and liver tumor cell lines. (A) Listing of cases; (B) Summary

**A**

| No. | Sex | G | Etiology | Gains | Losses |
|---|---|---|---|---|---|
| 1 | m | G1 | HBV | 1q, 2, 5p, 5q, 6p, 7p21-22, 7q, 8q21.1-21.3, 13q31-34, 17p | 3p, 6q, 12q15-24.3, 21q21-22 |
| 2 | m | G2 | ethanol | 1q12-25, 1p, 2p, 3q, 6p11-21.2, 9p, 17q, 19q12-13.2, 20q, Xq22-26 | 3p, 4q, 6q31-34, 8p, 9q, 10q 22-26, 13q, 16q, 20p, Y |
| 3 | m | G2 | HBV | – | – |
| 4 | f | G2-3 | hemochrom. | 8q24.2-24.3, 11q12-14 | 4q |
| 5 | m | G1 | Ø | 8q11.1-36, 15p, 15q11.1-12, 22p, 22q | – |
| 6 | m | G1 | Ø | 1q, 8q21.1-24.3, Xp, Xq21-28 | – |
| 7 | m | G3 | HBV | 1q | – |
| 8 | m | G3 | ethanol | 1q, 1p33-11, 4p16-14, 15q13-22, 17q | 4q24-25, 11q24-25, 13q, 14q33, 21q, Yq |
| 9 | m | G2 | ethanol | 1q | – |
| 10 | f | G1 | Ø | – | – |
| 11 | m | G1 | Ø | 14p13-q12, 15p13-q13 | – |
| 12 | m | G1 | HBV | 7p, Xq | 4q, 5p15.1-15.3, 5q11.2-21, 10q, 16q12.1-23 |
| 13 | m | G1 | Ø | 1q11-31, 17q | 16q23-24 |
| 14 | m | G2 | HBV | 1q, 6, 8q, 11p15, 13q33-34, 14q32, 15q11.2-15, 21 | 8p2-23, Y |
| 15 | m | G2-3 | HBV | 8q | Y |
| 16 | m | G2 | ethanol | – | – |
| 17 | m | G3 | Ø | 1p, 1q11-33, 3p11-14 3q, 4p, 6p12-21.3, 8q12-24.1, 12q, 17q, 20 | 3p22-26, 4q13-35, 6q13-27, 13q, 14q, Y11.2 |
| 18 | f | G1 | Ø | 1q12-32, 3, 5p, 5q11.1-14, 6p, 7p12-15, 8, 9q11-21, 17q, 18, 20, X | 4q, 11, 12p12-13, 16q21-24 |
| 19 | m | G2 | HBV | 8q, 17q21-25, 20q13.2-13.3 | Yq |
| 20 | m | G2 | HBV | 1q12-41, 6, 7p, 8q13-24.1, 20 | 4q, 8p, 12p, Y11.2 |
| 21 | f | G2-3 | Ø | 1q, 2q32-37, 7p14-22, 8q, 12q21-24.1, 13q31-34, 20q | 4p16, 4q22-28, 6q, 10q25-26, 13p, 13q11-14, 16p12-13.3, 21q, 22, X |
| | | * HEP-3B | | 1q, 3q21.6-29, 4p, 6p, 7p, 11p, 13qter, 19p, 20q | 4q24-q 35, 5q14-q23, 13q14-q22, Y |
| | | *SK-HEP1 | | 6p, 17 | 4, 6q, 9, 18q12-23, Y |
| | | *HEP-G2 | | 2, 6p, 16p, 16q, 17q, 19p, 20 | 13q14-22 |

*: cell line; G: grade; Ø: no defined etiology (HBV and haemochromatosis negative).

**B**

| | No. of cases | % | Genomic imbalance |
|---|---|---|---|
| Gains | 12 | 57 | 1q (core region: 1q12-25) |
| | 11 | 52 | 8q (core region: 8q21.2-24.3) |
| | 6 | 29 | 17q (core region: 17q21.3-25) |
| | 6 | 29 | 20q |
| | 5 | 24 | 7p |
| Losses | 7 | 33 | 4q (core region:4q22-28) |
| | 4 | 19 | 13q (core region:13q12-14) |
| | 4 | 19 | 6q |
| | 3 | 14 | 16q |

imbalances to co-segregate. There was no correlation of the most frequent imbalances (gains of 1q and 8q and losses of 4q and 13q) compared to the grade of tumour differentiation, with the exception of 13q losses that were not detected in highly differentiated (G1) HCCs.

Since gains of genomic material may correlate with activated oncogenes and losses with the inactivation of tumour suppressor genes, a database analysis (OMIM-NCBI) was performed in order to identify known genes with potential relevance for the tumorigenic process located in these chromosomal regions. A number of genes of specific interest in the context of HCC that were identified by this search are shown in Figure 1.

To further analyse the potential consequences of genomic imbalances on gene expression, cDNA array analyses (Clontech) were performed with tumourous and non-tumourous liver RNA from tumour No. 6 (with 1q; 8q and X gains). Of the 518 genes evaluated, 126 genes showed significantly altered (> 3-fold) expression composed to the non-tumourous liver tissue; of those, 8 genes located in these chromosomal regions showed significant

 *© 2001 Cancer Research Campaign*

BJOC 01-1963 697-704 20/8/01 3:25 pm Page 701

**Table 2** Interphase FISH analysis

| Tumour No. | Probes test/reference | Q ≤ 0.5 (%) | 1 > Q > 0.5 (%) | Q = 1 (%) | Q > 1 (%) |
|---|---|---|---|---|---|
| 8 | 13/10 (13q14(RB-1) / 10p11.1-q11.1 (α-sat. DNA)) | 83.3 | 10.2 | 5.6 | 0.9 |
| 6 | 1/17 (1q12 (sat. II and III) / chr.17p11.1-q11.1 (α-sat. DNA)) | 0 | 1.9 | 30.7 | 67.3 |
| | 8/10 (8p11.1-q11.1 (α-sat. DNA) / 10p11.1- q11.1 (α-sat. DNA)) | 1.8 | 10.7 | 40.2 | 47.3 |

Q: ratio of the numbers of signals per nucleus using the 2 indicated hybridization probes.

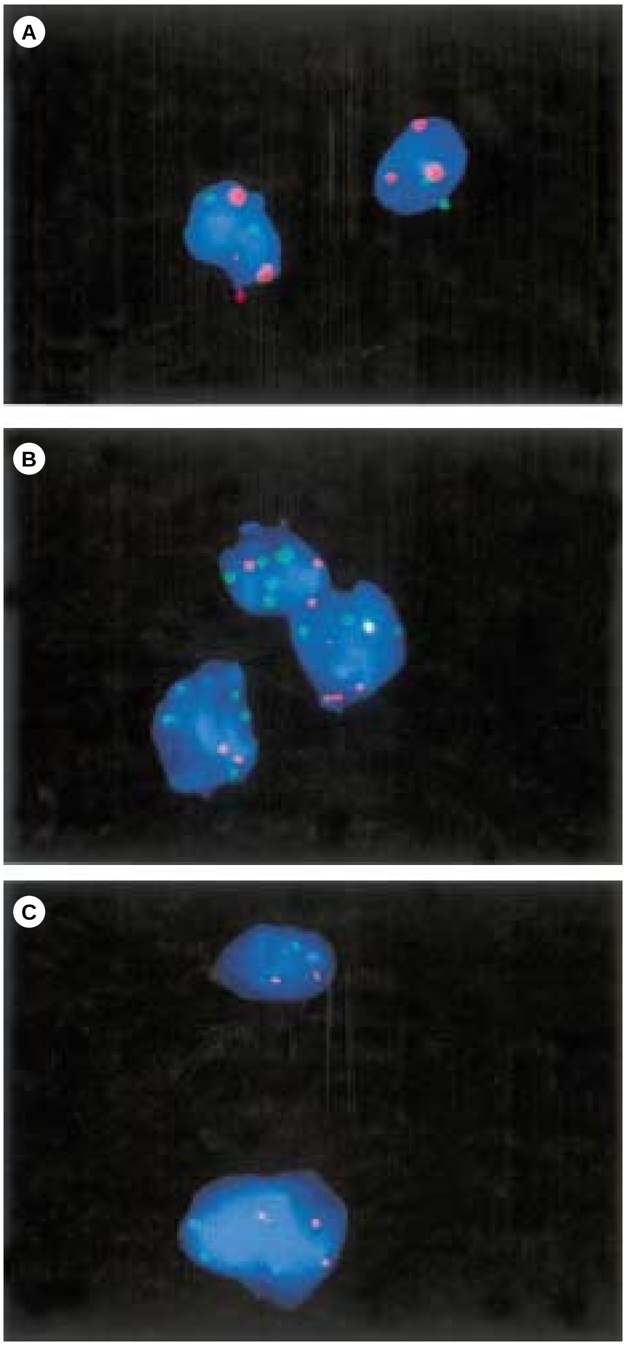

**Figure 2** Representative interphase FISH analyses in tumour Nos 6 and 8. (**A**) Interphase FISH with a satellite-DNA centromeric probe specific for chromosome 1 (red) and a satellite-DNA centromeric probe for chromosome 17 (green) (tumour No. 6). (**B**) Interphase FISH with a satellite-DNA-probe specific for chromosome 8 (green) and a satellite-DNA-probe specific for chromosome 10 (red) (tumour No. 6). (**C**) Interphase FISH with a LSI-probe for 13q14 covering the RB-1 gene (green) and a satellite-DNA-probe for chromosome 10 (red) (tumour No. 8).

overexpression in the tumour RNA (Table 3), among them the protooncogene c-*ski*.

### DISCUSSION

The present CGH study of human HCCs has identified a number of prominent genomic imbalances (Table 1 and Figure 1) that were selectively confirmed by FISH analyses of the respective interphase HCC nuclei. Our data show in part a correlation with one previous analysis (Marchio et al, 1997), although significant differences to this study exist. The overall percentage of genomic imbalances in our material is lower, which may be due to differences in case selection, not limited to HBV-induced HCCs in our study, but more likely to the stringent criteria applied to the acceptance of genomic imbalances in the present study. Some of the previously reported alterations (1p del, 4p del, 17p del, 19q amp, and X del) were not detected or did not reach significance in our analysis. In contrast there is a good match in the type of genomic imbalances of our study to a second, recent one (Wong et al, 1999) although the percentage of imbalances in our study is slightly but consistently lower for each single type of imbalance. It can therefore be speculated that the extent and type of genomic imbalances may be influenced by the underlying etiology of HCC.

Significant gains of genomic material have been found at 1q, 8q, 17q and 20q. These alterations appear to be selected and maintained during hepatocarcinogenesis and thus represent candidate regions for relevant proto-oncogenes or growth-promoting genes. Since 1q and 8q gains and 4q losses are detectable in highly and poorly differentiated tumours at an almost equal frequency, there is no evidence for a correlation with tumour cell differentiation. In the core region of 1q, the retinoic acid receptor gamma gene is located (1q22-23) that was originally cloned from an HCC and which is frequently overexpressed in HCCs (de The et al, 1987; Benbrook et al, 1988). Furthermore the c-*myc* gene (as well as the *myc*-activator) is located in 8q24, and is known to be activated in HCC cells (Huber and Thorgeirsson, 1987). The functional relevance of its overexpression due to genomic amplification is well documented (Koskinen and Alitalo, 1993). Correlative cDNA microarray expression screening in tumour No. 6 that showed gain

*© 2001 Cancer Research Campaign* 

BJOC 01-1963 697-704 20/8/01 3:25 pm Page 702

**Table 3** Overexpression of genes located in overrepresented chromosomal regions 1q and 8q (tumour No. 6)

| Gene | Chromosomal localization | Fold overexpression |
|---|---|---|
| Calgranulin (B) (MRP-14[calcium-binding protein in macrophages, MIF-related]) | 1q12-q22 | 3.5 |
| Interferon-gamma inducible protein | 1q22 | 3.1 |
| MNDA Myeloid cell nuclear differentiation antigen | 1q22 | * |
| *Ski* | 1q22-q24 | 8 |
| Transcription factor S-II-related protein (transcription elongation factor [SII]) | 8q | 27.8 |
| Interleukin-9-receptor | Xq28 | * |
| ARD (N-acetyltransferase ARDI) | Xq28 | 3.7 |
| Zinc finger x-chromosomal protein (ZFX putative transcription activator) | Xp22-p21.3 | * |

*: not evaluated due to lack of significant expression in non-tumourous tissue.

of 1q and 8q (see Table 1) has identified a number of genes that are highly overexpressed in the tumour tissue and are located in these chromosomal regions: (1q: *SKI*, 8q: *SII*) compared to the non-tumourous liver, suggesting that at least for some genes gains of genomic material may support overexpression. *SKI* appears particularly to be activated by overexpression and to represent a potential target gene since it has been recently shown to suppress transcriptional repression exerted by the Smad-mediated TGF-β activated pathway (Sun et al, 1999). This growth-suppressive pathway is deactivated at least in a wide variety of malignant epithelial tumours (Hata et al, 1998).

In 4 cases, deletions encompass 13q, a chromosomal region that contains not only the *RB*-1 (13q14.1-14.2), but also the *BRCA*-2 (13q12.3) gene. LOHs in 13q are relatively frequent in HCCs (up to 49%) and additionally sequence abnormalities in the *RB*-1 gene have been detected in up to 25% of HCCs (Murakami et al, 1991). Loss of function of the *BRCA*-2 gene has so far not been described in HCCs, but beside its well-documented role in breast cancer (Wooster et al, 1994, 1995), it also occurs in sporadic cancer of the exocrine pancreas (Schutte et al, 1995; Teng et al, 1996), an organ with its cellular origin embryologically closely related to hepatic tissue.

There are 3 cases that show deletions of 16q, which contain the cadherin gene cluster including E-cadherin at 16q22.1. Loss of E-cadherin expression has been linked to the gain of invasive potential (Frixen et al, 1991; Vleminckx et al, 1991; Berx et al, 1995) and has recently been identified as the cause of hereditary gastric cancer (Guilford et al, 1998). LOHs at 16q have on one hand been observed in 40–70% of the cases in HCC studies (Nishida et al, 1992; Yeh et al, 1996) but have conversely been correlated with advanced stages (Nishida et al, 1992) and dedifferentiated morphology of HCC (Shimoyama and Hirohashi, 1991). Functional inactivation of E-cadherin in carcinomas may additionally arise epigenetically through hypermethylation (Graff et al, 1995). Interestingly the Fanconi anemia gene is also located at 16q24.3 (Gschwend et al, 1996), and it may be hypothesized that in addition to loss of E-cadherin expression, somatic activation of a chromosomal breakage phenotype may support HCC progression. Another chromosomal breakage syndrome, ataxia teleangiectasia, may carry a slightly increased risk of hepatocellular carcinoma (Weinstein et al, 1985).

Chromosomal macrodeletions were most frequently found in 4q. A tumour-suppressor gene relevant for HCC development has already been suspected at this location from several LOH studies (frequency: 17–77%) (Buetow et al, 1989; Zhang et al, 1990; Konishi et al, 1993; Yeh et al, 1996; Chou et al, 1998). A common chromosomal breakpoint mapped to 4q has also been found in 4 out of 50 HCCs (Pasquinelli et al, 1988) as well as an HBV integration site in an HCC at 4q32.1. Furthermore caspase genes (*caspase*-6 (4q25), *caspase*-3 (4q35)) and the *smad*1 gene (4q28) are located at 4q. Nevertheless a definite target tumour-suppressor gene at 4q has so far not been identified.

In contrast other LOHs frequently observed at 17p (up to 64%), 1p (30–83%) and 11p (up to 44%) did not correlate with losses of genomic material in our CGH analysis and are thus unlikely to result from macrodeletions. Thus macrodeletions were not found to encompass the p53 locus at 17p13.1, in consistence with the fact that frequently the loss of p53 function in HCCs is due to point mutations at codon 249 (Fujimoto et al, 1994; Bressac et al, 1991), and not to deletions as described for glioblastomas (Albertoni et al, 1998). Furthermore no significant gains of genomic material have been found at chromosomal locations of growth factors that are frequently overexpressed in HCCs, such as IGF-II (11p15.5) and TGFα (2p13), suggesting that genomic amplification is a rather unlikely cause of growth factor overexpression in HCCs.

In conclusion, our CGH analysis of HCCs induces several hypotheses regarding activated or suppressed genes relevant to hepatocarcinogenesis. Some of them (RB-1 and E-cadherin) are reflected by preliminary expression data but many more including the ones mentioned events (Figure 1) will have to be tested by appropriate expression analyses.

### ACKNOWLEDGEMENTS

We thank K Petmecky for excellent technical assistance, as well as P Lichter, S Joos (DKFZ, Heidelberg, Germany), J. Ohlert and H. Müntefering (University of Mainz, Germany) for helpful advice. The study was supported by the Deutsche Forschungsgemeinschaft (projects A1 (PS) and C2 (HJD) of SFB 519), the Stiftung Rheinland Pfalz für Innovation (PS, HPD) and Köln-Fortune (PS).

### REFERENCES

Albertoni M, Daub DM, Arden KC, Viars CS, Powell C and Van Meir EG (1998) Genetic instability leads to loss of both p53 alleles in a human glioblastoma. *Oncogene* **16**: 321–326

Benbrook D, Lernhardt E and Pfahl M (1988) A new retinoic acid receptor identified from a hepatocellular carcinoma. *Nature* **333**: 669–672

Berx G, Cleton-Jansen AM, Nollet F, de Leeuw WJ, Van de Vijver M, Cornelisse C and Van Roy F (1995) E-cadherin is a tumor/invasion suppressor gene mutated in human lobular breast cancers. *EMBO J* **14**: 6107–6115

 © 2001 Cancer Research Campaign

BJOC 01-1963 697-704 20/8/01 3:25 pm Page 703

Bosch FX (1997) Global epidemiology of hepatocellular carcinoma. In: Okuda K, Tabor E. (eds) *Liver Cancer*. pp 13–28 Churchill Livingstone: New York, Edinburgh, London, Madrid, Melbourne, San Francisco, Tokyo

Bressac J, Kew M, Wands J and Ozturk M (1991) Selective G to T mutations of the p53 gene in hepatocellular carcinoma from southern Africa. *Nature* **350**: 429–431

Buetow KH, Murray JC, Israel JL, London WT, Smith M, Kew M, Blanquet V, Brechot C, Redeker A and Govindarajah S (1989) Loss of heterozygosity suggests tumor suppressor gene responsible for primary hepatocellular carcinoma. *Proc Natl Acad Sci USA* **86**: 8852–8856

Chou YH, Chung KC, Jeng LB, Chen TC and Liaw YF (1998) Frequent allelic loss on chromosomes 4q and 16q associated with human hepatocellular carcinoma in Taiwan. *Cancer Lett* **123**: 1–6

de la Coste A, Romagnolo B, Billuart P, Renard CA, Buendia MA, Soubrane O, Fabre M, Chelly J, Beldjord C, Kahn A and Perret C (1998) Somatic mutation of the beta-catenin gene are frequent in mouse and human hepatocellular carcinomas. *Proc Natl Acad Sci USA* **95**: 8847–8851

Deloukas P, Schuler GD, Gyapay G, Beasley EM, Soderlund C, Rodriguez-Tome P, Hui L, Matise TC, McKusick KB, Beckmann JS, Bentolila S, Bihoreau MT, Birren BB, Browne J, Butler A, Castle AB, Chiannilkulchai N, Clee C, Day PJR, Dehejian A, Dibling T, Drouot N, Duprat S, Fizames C, Fox S, Gelling S, Green L, Harrison P, Hocking R, Holloway E, Hunt S, Keil S, Lijnzaad P, Louis-Dit-Sully C, MaJMendis A, Miller J, Morissette J, Muselet D, Nusbaum HC, Peck A, Rozen S, Simon D, Slonim DK, Staples R, Stein DL, Stewart EA, Suchard MA, Thangarajah T, Vega-Czarny N, Webber C, Wu X, Hudson J, Auffray C, Nomura N, Sikela MJ, Polymeropoulos MH, James MR, Lander ES, Hudson TJ, Myers RM, Cox DR, Weissenbach J, Boguski MS and Bentley DR (1998) A physical map of 30,000 human genes. *Science* **282**: 744–746

Derynck R, Goeddel DV, Ullrich A, Gutterman JU, Williams RD, Bringman TS and Berger WH (1987) Synthesis of messenger RNAs for transforming growth factors alpha and beta and the epidermal growth factor receptor by human tumors. *Cancer Res* **47**: 707–712

de The H, Marchio A, Tiollais P and Dejean A (1987) A novel steroid thyroid hormone receptor-related gene inappropriately expressed in human hepatocellular carcinoma. *Nature* **330**: 667–670

du Manoir S, Schröck E, Bentz M, Speicher MR, Joos S, Ried T, Lichter P and Cremer T (1995) Quantitative analysis of comparative genomic hybridization. *Cytometry* **19**: 27–41

Fourel G, Trepo C, Bougueleret L, Henglein B, Ponzetto A, Tiollais P and Buendia MA (1990) Frequent activation of N-myc genes by hepadnavirus insertion in woodchuck liver tumors. *Nature* **347**: 294–298

Frixen UH, Behrens J, Sachs M, Eberle G, Voss B, Warda A, Lochner D and Birchmeier W (1991) E-cadherin-mediated cell-cell adhesion prevents invasiveness of human carcinoma cells. *J Cell Biol* **113**: 173–185

Fujimoto Y, Hampton LL, Wirth PJ, Wang NJ, Xie JP and Thorgeirsson SS (1994) Alterations of tumor suppressor genes and allelic losses in human hepatocellular carcinomas in China. *Cancer Res* **54**: 281–285

Geissler M, Gesien A and Wands JR (1997) Molecular mechanisms of hepato-carcinogenesis. In: Okuda K and Tabor E. (eds) *Liver Cancer*, pp 59–88. Churchill Livingstone: New York, Edinburgh, London, Madrid, Melbourne, San Francisco, Tokyo

Graff JR, Herman JG, Lapidus RG, Chopra H, Xu R, Jarrard DF, Isaacs WB, Pitha PM, Davidson NE and Baylin SB (1995) E-cadherin expression is silenced by DNA hypermethylation in human breast and prostate carcinomas. *Cancer Res* **55**: 5195–5199

Gschwend M, Levran O, Kruglyak L, Ranade K, Verlander PC, Shen S, Faure S, Weissenbach J, Altay C, Lander ES, Auerbach AD and Botstein D (1996) A locus for Fanconi anemia on 16q determined by homozygosity mapping. *Am J Hum Genet* **59**: 377–384

Guilford P, Hopkins J, Harraway J, McLeod M, McLeod N, Harawira P, Taita H, Scoular R, Miller A and Reeve AE (1998) E-cadherin germline mutations in familial gastric cancer. *Nature* **392**: 402–405

Hata A, Shi Y and Massague J (1998) TGF-beta signaling and cancer: structural and functional consequences of mutations in Smads. *Mol Med Today* **4**: 257–262

Hopman AHN, Poddighe P, Moesker O and Ramaekers FCS (1992) Interphase cytogenetics: an approach to the detection of genetic aberrations in tumors. In: Herrington CS, Mc Gee JO'D (eds), *Diagnostic Molecular Pathology A Practical Approach* pp 141–163

Hsu IC, Metcalf RA, Sun T, Welsh JA, Wang NJ and Harris CC (1991) Mutation hotspot in the p53 gene in human hepatocellular carcinomas. *Nature* **350**: 427–428

Huber BE and Thorgeirsson SS (1987) Analysis of c-myc expression in a human hepatoma cell line. *Cancer Res* **47**: 3414–3420

Ishak KG, Anthony PP and Sobin LH (1994) *Histological Typing of Tumors of the Liver*. p. 125 Springer: Berlin

Konishi M, Kikuchi-Yanoshita R, Tanaka K, Sato C, Tsuruta K, Maeda Y, Koike M, Tanaka S, Nakamura Y, Hattori N and Miyaki M (1993) Genetic changes and histopathological grades in human hepatocellular carcinomas. *Cancer Res* **84**: 893–899

Koskinen PJ and Alitalo K (1993) Role of myc amplification and overexpression in cell growth, differentiation and death. *Semin Cancer Biol* **4**: 3–12

Lichter P, Bentz M, du Manoir S and Joos S (1994) Comparative genomic hybridization. In: Verma R, Babu A (eds), *Human Chromosomes*, pp 191–210. Mc Graw-Hill: New York

Marchio A, Meddeb M, Pineau P, Danglot G, Tiollais P, Bernheim A and Dejean A (1997) Recurrent chromosomal abnormalities in hepatocellular carcinoma detected by comparative genomic hybridization. *Genes Chromosomes Cancer* **18**: 59–65

McMahon G, Davis EF, Huber LJ, Kim Y and Wogan GN (1990) Characterization of c-Ki-ras and N-ras oncogenes in aflatoxin B1-induced rat liver tumors. *Proc Natl Acad Sci USA* **87**: 1104–1108

Murakami Y, Hayashi Y, Hirohashi S and Sekiya T (1991) Aberrations of the tumor suppressor p53 and retinoblastoma genes in human hepatocellular carcinoma. *Cancer Res* **51**: 5520–5525

Nagai H, Pineau P, Tiollais P, Buendia MA and Dejean A (1997) Comprehensive allelotyping of hepatocellular carcinoma. *Oncogene* **14**: 2927–2933

Nishida N, Fukuda Y, Kokuryu H, Sedamoto T, Isowa G, Honda K, Yamaoka Y, Ikenaga M, Imura H and Ishizaki K (1992) Accumulation of allelic loss on arms of chromosome 13q, 16q and 17p in the advanced stages of human hepatocellular carcinoma. *Int J Cancer* **51**: 862–868

Nong Z, Siegel K, Odenthal M, Becker R, Oesch F, Dienes HP, Schirmacher P and Steinberg P (1997) The role of insulin-like growth factor II in the malignant transformation of liver oval cells. *Hepatology* **25**: 900–905

Pasquinelli C, Garreau F, Bougueleret L, Cariani E, Grzeschik KH, Thiers V, Croissant O, Hadchouel M, Tiollais P and Brechot C (1988) Rearrangement of a common cellular DNA domain on chromosome 4 in human liver tumors. *J Virol* **62**: 629–632

Sambrook J, Fritsch EF and Maniatis T (1989) Molecular cloning: *A laboratory manual*. Laboratory Press: Cold Spring Harbor

Schirmacher P, Held WA, Yang D, Chisari FV, Rustum Y and Rogler CE (1992) Reactivation of insulin-like growth factor II during hepatocarcinogenesis in transgenic mice suggests a role in malignant growth. *Cancer Res* **52**: 2549–2556

Schirmacher P and Dienes HP (1999) Hepatocellular carcinoma. In: Kurzrock R, Talpaz M (eds), *Molecular Biology in Cancer Medicine*. pp 355–366. Martin Dunitz: London, Malden, Winnipeg, Sao Paulo

Schutte M, da Costa LT, Hahn SA, Moskaluk C, Hoque ATMS, Rozenblum E, Weinstein CL, Bittner M, Meltzer PS, Trent JM, Yeo CJ, Hruban RH and Kern SE (1995) Identification by representational difference analysis of a homozygous deletion in pancreatic carcinoma that lies within the BRCA2 region. *Proc Natl Acad Sci USA* **92**: 5950–5954

Sinha S, Webber C, Marshall CJ, Knowles MA, Proctor A, Barrass NC and Neal GE (1988) Activation of ras oncogene in aflatoxin-induced rat liver carcinogenesis. *Proc Natl Acad Sci USA* **85**: 3673–3677

Shimoyama Y and Hirohashi S (1991) Cadherin intercellular adhesion molecule in hepatocellular carcinomas: loss of E-cadherin expression in an undifferentiated carcinoma. *Cancer Lett* **57**: 131–135

Sun Y, Lix X, Eaton EN, Lane WS, Lodish HF and Weinberg RA (1999) Interaction of the Ski oncoprotein with Smad3 regulates TGF-beta signaling. *Mol Cell* **4**: 499–509

Teng DHF, Bogden R, Mitchell J, Baumgard MII R, Berry S, David T, Ha PC, Kehrer R, Jammulapati S, Chen Q, Offit K, Skolnick MH, Tavtigian SV, Jhanwar S, Swedlund B, Wong AKC and Kamb A (1996) Low incidence of BRCA2 mutations in breast carcinoma and other cancers. *Nature Genet* **13**: 241–244

Tsuda H, Zhang W, Shimosato Y, Yokota J, Terada M, Sugimura T, Miyamura T and Hirohashi S (1990) Allele loss on chromosome 16 associated with progression of human hepatocellular carcinoma. *Proc Natl Acad Sci USA* **87**: 6791–6794

Tsuda H, Oda T, Sakamoto M and Hirohashi S (1992) Different pattern of chromosomal allele loss in multiple hepatocellular carcinomas as evidence of their multifocal origin. *Cancer Res* **52**: 1504–1509

Vleminckx K, Vakaet L, Mareel M, Fiers W and Roy FV (1991) Genetic manipulation of E-cadherin expression by epithelial tumour cells reveals an invasion suppressor role. *Cell* **66**: 107–119

Vogel W and Speit G (1985) Zytogenetik. In: Wolf U and Winkler U (eds), *Humangenetik*. pp 10–16. Springer-Verlag: Berlin, Heidelberg, New York, Tokyo

Walker GJ, Hayward NK, Falvey S and Cooksely WG (1991) Loss of somatic heterozygosity in hepatocellular carcinoma. *Cancer Res* **51**: 4367–4370

*© 2001 Cancer Research Campaign* 

Weinstein S, Scottalini AG, Loo SYT, Caldwell PC and Bhagavan NV (1995) Ataxia teleangiectasia with hepatocellular carcinoma in a 15 year old girl and studies of her kindred. *Arch Pathol Lab Med* **109**: 1000–1004 (1985)

Wong N, Lai P, Lee SW, Fan S, Pang E, Liew CT, Sheng Z, Lau JW and Janson PJ (1999) Assessment of genetic changes in hepatocellular carcinoma by comparative genomic hybridization analyses: relationship to disease stage, tumor size, and cirrhosis. *Am J Pathol* **154**: 37–43

Wooster R, Neuhausen SL, Mangion J, Quirk Y, Ford D, Collins N, Nguyen K, Seal Tran T, Averill D, Fields P, Marshall G, Narod S, Lenoir GM, Lynch H, Feunteun J, Devilee P, Cornelisse CJ, Menko FH, Daly PA, Ormiston W, McManus R, Pye C, Lewis CM, Cannon-Albright LA, Peto J, Ponder BAJ, Skolnick MH, Easton DF, Goldgar DE and Stratton MR (1994) Localization of a breast cancer susceptibility gene, BRCA2, to chromosome 13q12–13. *Science* **265**: 2088–2090

Wooster R, Bignell G, Lancaster J, Swift S, Seal S, Mangion J, Collins N, Gregory S, Gumbs C, Micklem G, Barfoot R, Hamoudi R, Patel S, Rice C, Biggs P, Hashim Y, Smith A, Connor F, Arason A, Gudmundsson J, Ficenec D, Keisell D, Ford D, Tonin P, Biship DT, Spurr NK, Ponder BAJ, Eeles R, Peto J, Devilee P, Cornelisse C, Lynch H, Narod S, Lenoir G, Egilsson V, Barkadottir RB, Easton DF, Bentley DR, Futreal PA, Ashworth A and Stratton MR (1995) Identification of the breast cancer susceptibility gene BRCA2. *Nature* **378**: 789–792

Yamada T, De Souza AT, Finkelstein S and Jirtle RL (1997) Loss of the gene encoding mannose 6-phosphate/insulin-like growth factor II receptor is an early event in liver carcinogenesis. *Proc Natl Acad Sci USA* **94**: 10351–10355

Yeh SH, Chen PJ, Lai MY and Chen DS (1996) Allelic loss on chromosomes 4q and 16q in hepatocellular carcinoma: association with elevated $\alpha$-fetoprotein. *Gastroenterology* **110**: 184–192

Zhang W, Hirohashi S, Tsuda H, Shimosato Y, Yokota J, Terada M and Sugimura T (1990) Frequent loss of heterozygosity on chromosomes 16 and 4 in human hepatocellular carcinoma. *Jpn J Cancer Res* **81**: 108–111

 *© 2001 Cancer Research Campaign*
